# Quality of care assessment for non-small cell lung cancer patients: transforming routine care data into a continuous improvement system

**DOI:** 10.1007/s12094-024-03658-3

**Published:** 2024-08-16

**Authors:** Juan C. Sánchez, Beatriz Nuñez-García, Yago Garitaonaindia, Virginia Calvo, Mariola Blanco, Arturo Ramos Martín-Vegue, Ana Royuela, Marta Manso, Blanca Cantos, Miriam Méndez, Ana Collazo-Lorduy, Mariano Provencio

**Affiliations:** 1 Medical Oncology Department, Puerta de Hierro-Majadahonda University Hospital, C. Joaquín Rodrigo, 1, Majadahonda, 28222 Madrid, Spain; 2 Admission and Clinical Documentation Department, Puerta de Hierro-Majadahonda University Hospital, Madrid, Spain; 3 Biostatistics Unit, Hospital Universitario Puerta de Hierro Majadahonda, IDIPHISA. CIBERESP, ISCIII, Madrid, Spain; 4 Hospital Pharmacy Service, Puerta de Hierro-Majadahonda University Hospital, Madrid, Spain

**Keywords:** Quality of care, Quality indicators, Lung neoplasms, Real world data, Quality improvement

## Abstract

**Purpose:**

The complexity of cancer care requires planning and analysis to achieve the highest level of quality. We aim to measure the quality of care provided to patients with non-small cell lung cancer (NSCLC) using the data contained in the hospital’s information systems, in order to establish a system of continuous quality improvement.

**Methods/Patients:**

Retrospective observational cohort study conducted in a university hospital in Spain, consecutively including all patients with NSCLC treated between 2016 and 2020. A total of 34 quality indicators were selected based on a literature review and clinical practice guideline recommendations, covering care processes, timeliness, and outcomes. Applying data science methods, an analysis algorithm, based on clinical guideline recommendations, was set up to integrate activity and administrative data extracted from the Electronic Patient Record along with clinical data from a lung cancer registry.

**Results:**

Through data generated in routine practice, it has been feasible to reconstruct the therapeutic trajectory and automatically calculate quality indicators using an algorithm based on clinical practice guidelines. Process indicators revealed high adherence to guideline recommendations, and outcome indicators showed favorable survival rates compared to previous data.

**Conclusions:**

Our study proposes a methodology to take advantage of the data contained in hospital information sources, allowing feedback and repeated measurement over time, developing a tool to understand quality metrics in accordance with evidence-based recommendations, ultimately seeking a system of continuous improvement of the quality of health care.

**Supplementary Information:**

The online version contains supplementary material available at 10.1007/s12094-024-03658-3.

## Introduction

Lung cancer, with 2.2 million new diagnoses and 1.8 million deaths per year worldwide, is the leading cause of cancer mortality, responsible for 18% of all cancer deaths [1]. In recent decades, advances in molecular biology, targeted therapies, and new treatments such as immunotherapy have increased lung cancer survival [2, 3]. To ensure quality care, we need to know how we treat our patients and measure how advances in research translate into better outcomes for the general population [4].

In recent years, healthcare systems have undergone rapid digital transformation, resulting in vast amounts of data emerging from the patient's medical care journey [5]. As a result, there is a growing interest in using real-world data to address relevant clinical and health policy questions that cannot be answered by clinical trials, including the assessment of quality of care [6].

The advancement of oncology has outpaced the slow development of process and outcome assessment [7] and the challenge of integrating quality assessment into health systems lies not so much in defining a set of indicators as in developing timely measurement tools and articulating them towards a quality improvement system. One solution to reduce administrative burden and ensure feasible assessment is automated extraction of data from existing sources, such as building measurement systems through feedback from the electronic patient record (EPR) [8, 9].

We aim to measure the quality of care provided to patients with non-small cell lung cancer (NSCLC) using the data contained in the hospital’s information systems, while establishing a quality improvement monitoring system. Applying data science methods, we set up an analysis algorithm, based on recommendations from clinical guidelines, to integrate activity and administrative data extracted from the EPR together with clinical data from a lung cancer registry in order to reconstruct the therapeutic trajectory and calculate quality indicators (QIs) that cover the overall care.

## Methods

### Design

This retrospective observational cohort study that included all patients with NSCLC treated by the Medical Oncology department at Puerta de Hierro University Hospital (HUPH), with a reference population of 500,000 inhabitants, between January 1, 2016 and December 31, 2020. The final follow-up date was June 30, 2021. The study protocol was approved by the HUPH Institutional Ethics Board.

### Selection of quality indicators

A literature review of “quality indicators in lung cáncer” was conducted based on a PubMed search, from 2005 to 2023. An evaluation of clinical guidelines was performed at NSCLC. Previously published recommended indicator characteristics were considered, including scientific evidence, development method, feasibility of calculation, and ability to discriminate differences [4, 10]. We selected QIs that correlated with recommendations from clinical practice guidelines, as previously described in NSCLC [8, 11–13] and calculable with the available data. A total of 34 QIs were selected to assess quality of care: 17 process indicators, 6 process indicators assessing the timeliness of care, and 11 outcome indicators.

### Data sources and calculation of quality indicators

Patients were identified from the HUPH tumor registry. Data obtained from the tumor registry were cross-referenced, using the hospital identification number, with a cancer repository created by the Medical Oncology department reviewing individual patient records, together with administrative data extracted from the EPR (Supplementary Material), linking all the available information of each patient and resulting in a new database that included demographic variables (age, gender), clinical (smoking history, CHARLSON comorbidity score, Eastern Cooperative Oncology Group score (ECOG)), diagnostic and pathological variables (date of diagnosis, histology, stage, molecular studies), as well as therapeutic information (surgical treatments, radiotherapy and medical treatments received with their related dates) and health status at the last follow-up. Healthcare data was obtained through Oracle Business Intelligence Discoverer 10 g Version 2 (10.1.2.1). Stata v.16 was used for data preparation, cross-referencing and depuration.

The Cross-Industry Standard Process for Data Mining *(CRISP-DM)* methodology was applied to achieve our goal [14], adapting this technique to analyze integrated clinical and healthcare usage data. Guideline-based decision trees were used to model and describe the patient's journey in healthcare based on the available data, while performing the calculation of the indicators.

### Statistical analysis

The categorical variables are described by absolute and relative frequencies, and for the numerical variables, we used the mean (standard deviation) or median (percentiles 25 and 75). Comparisons between categorical variables were performed the Pearson chi-squared test. A *p* value < 0.05 was considered statistically significant.

In the survival analyses, the Kaplan–Meier method and the log-rank test were used to compare the survival curves, with a *p*-value of < 0.05 considered significant. For multivariable analysis, a Cox proportional hazards regression model was used to evaluate the association of potential prognostic variables. All potential prognostic variables were included in the model, and then a backward selection strategy was carried out using the likelihood ratio test and a significance level of 0.05. Statistical analysis was performed using Stata v.16.

## Results

### Patient characteristics

Between January 1, 2016 and December 31, 2020, 821 lung cancer patients were identified in the HUPH tumor registry; 650 patients with NSCLC were consecutively included; the median follow-up was 37.6 months (95% CI: 33.4–41). The median age was 67 years (interquartile range (IQR): 60–74), 66.5% of patients were men; 89.6% were smokers or former smokers. The distribution of histological subtypes was as follows: 61.1% adenocarcinoma, 28% squamous cell carcinoma, 2.8% large cell carcinoma, and 8.1% other histology or carcinoma not otherwise specified. The characteristics of the population are described in Table 1. The treatments administered during the study period for each stage, also considering the presence of targeted mutations in advanced disease, are described in Table 2.

**Table 1:** Population characteristics

NSCLC	IA-IIB	IIIA-IIIC	IVA-IVB	Total NSCLC	*p*	Missing data
Patients: n	84	160	406	650		0%
Histology: n (%)					** < *0.001***	0%
Adenocarcinoma	47 (55.9%)	79 (49.4%)	271 (66.7%)	397 (61.1%)		
Squamous cell carcinoma	31 (38.1%)	68 (42.5%)	82 (20.2%)	182 (28%)		
Large cell carcinoma	2 (2.4%)	3 (1.9%)	13 (3.2%)	18 (2.8%)		
Not otherwise specified/Others	3 (3.6%)	10 (6.2%)	40 (9.9%)	53 (8.1%)		
Age: median (IQR)	69 (62 – 73.5)	67 (60 – 73.5)	67 (60–74)	67 (60–74)	**–**	0%
< 50 years	2.4%	5%	5.9%	5.2%	*0.865*	
50–64 years	34.5%	37.5%	33.7%	34.8%		
65–74 years	40.5%	35.6%	38.2%	37.8%		
≥ 75 years	22.6%	21.9%	22.2%	22.2%		
Sex: n (%)					*0.539*	0%
Female	26 (31%)	46 (28.8%)	136 (33.5%)	208 (32%)		
Male	58 (69%)	114 (71.2%)	270 (66.5%)	442 (66.5%)		
Smoking history: n (%)					*0.025*	0.6%
Never smoker	10 (12.2%)	9 (5.6%)	48 (11.9%)	67 (10.4%)		
Former smoker	45 (54.9%)	71 (44.4%)	201 (49.7%)	317 (49.1%)		
Active smoker	27 (32.9%)	80 (50%)	155 (38.4%)	262 (40.6%)		
Pack-year index: medina (IQR)	52 (39.5–70)	45 (36 – 62)	44 (30–62)	45 (31.5–65)	**–**	15.7%
Charlson comorbidity index: median (IQR)	6 (4–7)	5 (4 – 6.5)	9 (8–10)	8 (6–10)	**–**	0.2%
TNM 8th Stage: n (%)						0%
IA	18 (21.4%)			18 (2.8%)		
IB	22 (26.2%)			22 (3.4%)		
IIA	15 (17.9%)			15 (2.3%)		
IIB	29 (34.5%)			29 (4.5%)		
IIIA		94 (58.7%)		94 (14.4%)		
IIIB		52 (32.5%)		52 (8%)		
IIIC		14 (8.8%)		14 (2.1%)		
IVA			148 (36.5%)	148 (22.8%)		
IVB			258 (63.5%)	258 (39.7%)		
MOLECULAR PROFILE PERFORMED: n (%)			309 (76.1%)	376 (57.9%)		0%
EGFR +			50 (12.3%)	64 (9.9%)		
ALK +			12 (3%)	15 (2.3%)		
ROS1 +			0 (0%)	0 (0%)		
BRAF +			3 (0.7%)	4 (0.6%)		
PDL1 ASSESSMENT: n(%)						0%
PDL1 not performed			157 (38.7%)	320 (49.2%)		
PDL1 TPS 0			109 (26.8%)	147 (22.6%)		
PDL1 TPS 1 – 49			66 (16.3%)	86 (13.3%)		
PDL1 TPS > 49			74 (18.2%)	97 (14.9%)		
ECOG: n (%)					** < *0.001***	0.8%
0	46 (56.1%)	51 (32.3%)	49 (12.1%)	146 (22.6%)		
1	32 (39%)	85 (53.8%)	184 (45.4%)	301 (46.7%)		
2	4 (4.9%)	16 (10.1%)	95 (23.5%)	115 (17.8%)		
3	0	6 (3.8%)	70 (17.3%)	76 (11.8%)		
4	0	0	7 (1.7%)	7 (1.1%)		

*NSCLC* non-small cell lung cancer

*Smoking status was defined as: never smoker (< 100 cigarettes per lifetime); former smoker (100 cigarettes per lifetime and quit > 1 year prior to diagnosis); active smoker (100 cigarettes per lifetime and smoked at the time of lung cancer diagnosis or quit 1 year prior to the diagnosis)

**Table 2:** Therapeutic strategies used in nsclc by stage

Stage IA-IIB NSCLC					
TNM stage	IA	IB	IIA	IIB	Total NSCLC I-II
Patients: n	18	22	15	29	84
Treatment used: n (%)					
Surgery	17 (94.4%)	18 (81.8%)	15 (100%)	27 (93.1%)	77 (91.7%)
Pneumonectomy	0 (0%)	0 (0%)	1 (6.7%)	1 (3.5%)	2 (2.4%)
Neoadjuvant chemotherapy	0 (0%)	0 (0%)	0 (0%)	3 (10.3%)	3 (3.6%)
Adjuvant chemotherapy	0 (0%)	4 (18.2%)	10 (66.7%)	20 (69%)	34 (40.5%)
Adjuvant radiotherapy	0 (0%)	0 (0%)	0 (0%)	3 (10.3%)	3 (3.6%)
Adj. Chemo-radiotherapy	3 (16.7%)	0 (0%)	0 (0%)	0 (0%)	3 (3.6%)
Radiotherapy without surgery	1 (5.6%)	3 (13.6%)	0 (0%)	1 (3.5%)	5 (6%)
Best supportive care	0 (0%)	1 (4.6%)	0 (0%)	1 (3.5%)	2 (2.4%)

Stage IIIA–IIIC NSCLC				
TNM stage	IIIA	IIIB	IIIC	Total NSCLC III
Patients: n	94	52	14	160
Treatment used: n (%)				
Surgery	60 (63.8%)	6 (11.5%)	0 (0%)	66 (41.3%)
Pneumonectomy	5 (5.3%)	2 (3.9%)	0 (0%)	7 (4.4%)
Neoadjuvant therapy	51 (54.3%)	40 (76.9%)	11 (78.6%)	102 (63.8%)
Adjuvant therapy	35 (37.2%)	4 (7.7%)	5 (35.7%)	44 (27.5%)
Systemic treat. (any modality)	81 (86.2%)	47 (90.4%)	12 (85.7%)	140 (87.5%)
Clinical trial	21 (22.3%)	4 (7.7%)	0 (0%)	25 (15.6%)
RT or ChRT with no surgery	31 (33%)	35 (67.3%)	12 (85.7%)	78 (48.8%)
Adjuvant RT or ChRT	19 (20.2%)	3 (5.8%)	0 (0%)	22 (13.8%)
Best supportive care	2 (2.1%)	3 (5.8%)	1 (7.1%)	6 (3.8%)

Stage IVA–IVB NSCLC			
Presence of driver mutation	IV without targeted mutation	IV with targeted mutation	Total NSCLC IV
Patients: n	342	64	406
Systemic treat. (any modality)	251 (73.4%)	62 (96.1%)	313 (77.1%)
Paliative radiotherapy	178 (52.1%)	28 (43.8%)	206 (50.7%)
Best supportive care	91 (26.6%)	2 (3.1%)	93 (22.9%)
Number of lines: median (range)	2 (1–5)	2 (1–6)	2 (1–5)
First line: n (% among treated patients)	251 treated patients	62 treated patients	
Platinum doublet	164 (65.3%)	6 (9.7%)	
Immunotherapy	51 (20.4%)	1 (1.6%)	
Chemo-immunotherapy	21 (8.4%)	1 (1.6%)	
Targeted therapy	0 (0%)	54 (87.1%)	
1st line clinical trial: n (%)	49 (19.5%)	2 (3.2%)	
Any line: n (% among treated patients)	251 treated patients	62 treated patients	
Platinum doublet	201 (80.1%)	22 (35.5%)	
I-therapy or Ch-Immunotherapy	148 (59%)	8 (12.9%)	
Targeted therapy	0 (0%)	59 (95.2%)	
Any line clinical trial: n (%)	64 (25.5%)	13 (21%)	

*Adj* adjuvant, *Ch-Immunotherapy* chemotherapy and immunotherapy, *ChRT* chemotherapy and radiotherapy, *I-therapy* immunotherapy, *RT* radiotherapy

### Quality of care indicators

#### Analysis algorithm configuration

**Fig. 1:**
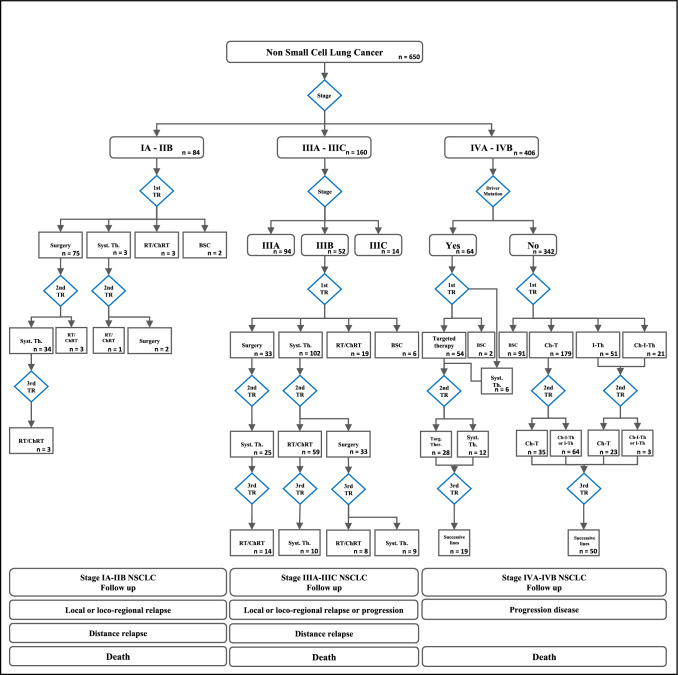
Therapeutic journey of the NSCLC cohort reconstructed using decision tres. BSC: best supportive care; Ch-I-Th: chemotherapy + immunotherapy; Ch-T: chemotherapy; I-Th: immunotherapy; RT/ChRT: radiotherapy/chemotherapy + radiotherapy; Syst Th: systemic therapy; TR: treatment,

An algorithm was constructed according to the recommendations of clinical guidelines to reconstruct the trajectory of care for the population of patients with NSCLC from EPR dataset. Illustrated as a decision tree in Fig. 1, it defines therapeutic pathways according to the value of key variables: stage, presence of targeted mutations, and treatments performed. The applied algorithm establishes the relationship between the data that allows the automated calculation of QIs from the hospital's information systems, Table 3, identifying process, timelines of care and result indicators to fully cover the care provided.

**Fig. 2:**
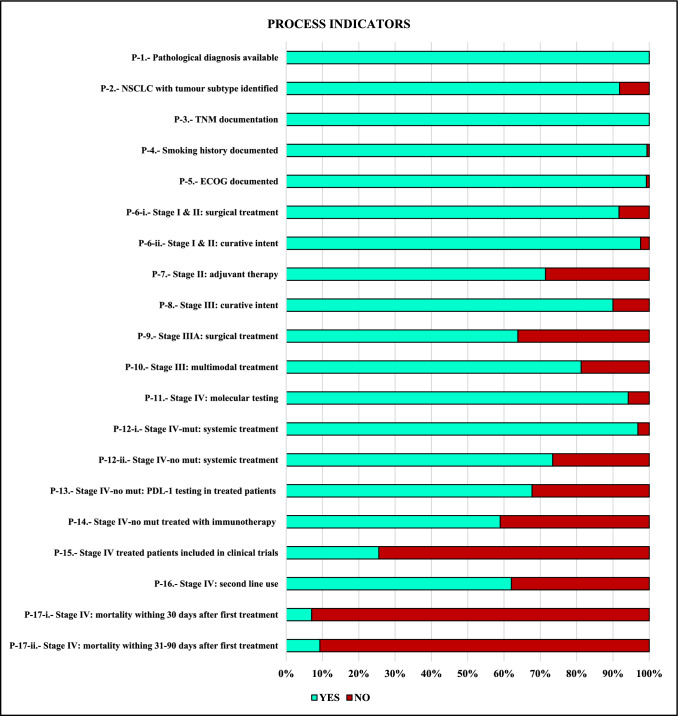
Selected process indicators for patient care in NSCLC. NSCLC: non small cell llung cancer; mut: mutated

**Fig. 3:**
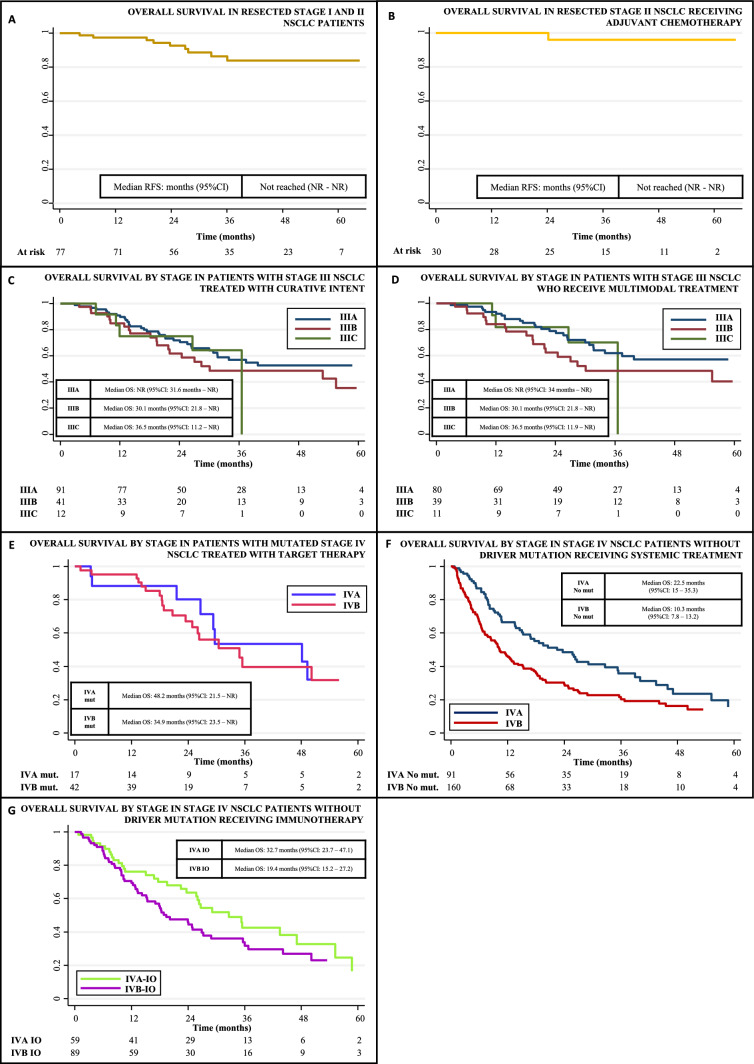
Outcome indicators for NSCLC. Kaplan–Meier curves of overall survival for patients with resected stage I and II (A), resected stage II receiving chemotherapy (B), stage III treated with curative intention, by stage (C), stage III who received multimodal treatment, by stage (D), mutated stage IV NSCLC treated with targeted therapy (E), stage IV NSCLC patients without driver mutation receiving systemic treatment, by stage (F) and stage IV NSCLC patients without driver mutation receiving immunotherapy, by stage (G)

- Calculated process indicators assess adherence to clinical guideline recommendations, Fig. 2. 91.7% of patients in stages I and II undergone surgery, 97.6% received treatment with radical intent. In addition, 71.4% of resected stage II patients received adjuvant chemotherapy. In stage III, 90% received treatment with curative intent, 87.5% received systemic treatment, and 81.3% completed a multimodal strategy. Regarding stage IIIA, 63.8% were treated with surgery. In advanced disease, 73.4% of non-mutated patients and 96.9% of mutated patients received systemic therapy. 24.5% participated in a clinical trial. The incorporation of immunotherapy into clinical practice in stage IV without targeted mutation was also evaluated, with a progressive increase in its use from 40.3% in 2016 to 71.2% in 2020. As part of process evaluation, it was possible to evaluate the timeliness of the correct care previously described, Table 3.
- Relapse-free survival (RFS), time to treatment failure (TTF) and overall survival (OS) in patients treated according to stage were analyzed as outcome indicators, Table 3 and Fig. 3, making it possible to know the outcome in the general population of the application of the treatments corresponding to each indication. In stage III patients treated with curative intent, the median OS was 52.8 months (95% CI: 30.1—not reached (NR)); In the case of stage IV systemic therapy, median OS was 34.9 months (95% CI: 26–50.1) for mutated patients and 13.6 months (95% CI: 10.5–17.9) for patients without mutation. In addition, the subgroup of 148 stage IV patients without targeted mutation treated with immunotherapy had a median OS of 25.7 months (95% CI: 18.9–32.7). Median OS was not reached in stages I-II or IIIA. An exploratory multivariate analysis was performed in the survival analysis including the variables: stage, histology, ECOG, sex and smoking history, observing an association between the risk of death and ECOG in the different indicators, as well as an increased risk of death for stage IVB compared to stage IVA and the histological subtype squamous cell carcinoma compared to adenocarcinoma in patients without targeted mutation.
- Diagnostic process indicators. To ensure the correct classification of each patient to their corresponding therapeutic route by the algorithm used, process indicators related to diagnosis were included, observing that 100% of the patients obtained a histological diagnosis, in 91.9% the histological subtype was identified and 100% of the patients had a documented tumor stage. In stage IV, molecular profiling (gene testing for EGFR, ALK, ROS1 and BRAF) was performed in 94.3% of patients with adenocarcinoma, non-specific carcinoma, or nonsmoking squamous cell carcinoma. In patients treated in stage IV without mutation, PDL-1 was determined in 67.7%, showing a progressive increase from 25.8% in 2016 to 96.2% in 2020.

**Table 3:** Quality indicators for NSCLC

Process of care						
Code	Indicator	Numerator	Denominator	Eligible Patients: n	Missing data: n(%)	Quality indicator result
All lung cancer patients						
P-1	Proportion of patients with histological study	Number of patients with lung cancer who have a pathological diagnosis	All patients with lung cancer	821	0 (0%)	100%
All NSCLC patients						
P-2	Proportion of patients with NSCLC who have tumour subtype identified	Number of patients with a pathological diagnosis of NSCLC who have tumour subtype identified	All patients with a pathological diagnosis of NSCLC	650	0 (0%)	91.9%
P-3	Proportion of patients with documented TNM stage	Number of patients with documented TNM stage	All NSCLC patients	650	0 (0%)	100%
P-4	Proportion of patients with a documented history of tobacco use	Number of patients with a documented history of tobacco use	All NSCLC patients	650	4 (0.6%)	99.4%
P-5	Proportion of patients with an assessment of functional status according to the ECOG scale	Number of patients with ECOG scale documented at the first consultation with medical oncology	All NSCLC patients	650	5 (0.8%)	99.2%
Stage IA–IIB NSCLC						
P-6	Proportion of patients who undergo surgical intervention (i) and proportion of patients who undergo any radical treatment (ii)	Number of stage IA-IIB NSCLC patients who undergo surgery (i). Number of patients who undergo any radical treatment.(surgery or radiotherapy) (ii)	All stage IA-IIB NSCLC patients	84	i: 0 (0%) ii: 0 (0%)	i: 91.7% ii: 97.6%
P-7	Proportion of patients with stage II NSCLC who receive adjuvant chemotherapy	Number of stage II NSCLC patients who undergo surgery and receive adjuvant chemotherapy	Number of stage II NSCLC patients who undergo surgery	42	0 (0%)	71.4%
Stage IIIA–IIIC NSCLC						
P-8	Proportion of patients who undergo any radical treatment (surgery or radiotherapy)	Number of stage III NSCLC patients who undergo any radical treatment.(surgery or radiotherapy)	All stage III NSCLC patients	160	0 (0%)	90%
P-9	Proportion of stage IIIA NSCLC patients who undergo surgical intervention	Number of stage IIIA NSCLC patients who undergo surgery with curative intent	All stage IIIA NSCLC patients	94	0 (0%)	63.8%
P-10	Proportion of patients with stage III NSCLC who receive multimodal treatment including systemic chemotherapy	Number of stage III NSCLC patients treated with systemic therapy and at least one radical treatment modality (surgery and/or radiotherapy)	All stage III NSCLC patients	160	0 (0%)	81.3%
Stage IVA–IVB NSCLC						
P-11	Proportion of patients with molecular testing performed	Number of patients diagnosed with adenocarcinoma, unspecified carcinoma, or non-smoker squamous cell carcinoma, stage IV, with molecular testing performed	All stage IV NSCLC patients and any of histology subtypes described in the numerator	328	0 (0%)	94.3%
P-12	Proportion of patients receiving systemic treatment: among those with driver mutation (i) and without driver mutation (ii)	Number of stage IV NSCLC patients receiving systemic treatment: with driver mutation (i) and without driver mutation (ii)	i: All stage IV NSCLC patients with driver mutation ii: without driver mutation	i: 64 ii: 342	i: 0 (0%) ii: 0 (0%)	i: 96.9% ii: 73.4%
P-13	Proportion of patients without mutation undergoing PDL1 testing among those receiving systemic treatment	Number of stage IV NSCLC patients without driver mutation who receive systemic treatment undergoing PDL1 testing	All stage IV NSCLC patients without driver mutation who receive systemic treatment	251	0 (0%)	Total: 67.7% Year 2016: 25.8% Year 2020: 96.2%
P-14	Proportion of patients without targetable mutations treated with immunotherapy or chemoimmunotherapy	Number of stage IV NSCLC patients without driver mutation treated with immunotherapy or chemoimmunotherapy	All stage IV NSCLC patients without driver mutation who receive systemic treatment	251	0 (0%)	Total: 59% Year 2016: 40.3% Year 2020: 71.2%
P-15	Proportion of patients who participated in a clinical trial among those who received systemic treatment	Number of stage IV NSCLC patients receiving systemic therapy as part of a clinical trial	All stage IV NSCLC patients receiving systemic therapy	313	0 (0%)	24.6%
P-16	Proportion of treated patients who receive a second line of treatment	Number of stage IV NSCLC patients who receive a second line of treatment	Number of stage IV NSCLC patients who progressed on or died during first line	266	0 (0%)	62%
P-17	Mortality within the first 30 days and the first 90 days after the start of systemic treatment	Number of patients with stage IV NSCLC who die within the first 30 (i) and 90 (ii) days after the start of systemic treatment	Number of stage IV NSCLC patients receiving any systemic treatment	313	0 (0%)	i: 7% ii: 9,3%

Timeliness of care						
Code	Indicator	Eligibility criteria	Outcome measure	Eligible Patients: n	Missing data: n(%)	Quality indicator result
Stage IA–IIB NSCLC						
T-1	Days between pathological diagnosis and surgical treatment	Patients with NSCLC stages IA to IIB surgically treated with curative intent	Median (days) and interquartile range	77	0 (0%)	Median: 33 days IQR: 0—50
T-2	Days between surgery and adjuvant chemotherapy (stage I-II NSCLC)	Patients with NSCLC stages IB to IIB who are surgically treated and receive adjuvant chemotherapy	Median (days) and interquartile range	34	1 (2.9%)	Median: 40 days IQR: 33—45
Stage IIIA–IIIC NSCLC						
T-3	Days between pathological diagnosis and first treatment (any modality) (stage III NSCLC)	Patients with NSCLC stage III receiving any treatment	Median (days) and interquartile range	154	0 (0%)	Median: 26 days IQR: 18.5 – 39.5
T-4	Days between surgery and adjuvant chemotherapy (stage III NSCLC)	Patients with NSCLC stage III who are surgically treated and receive adjuvant chemotherapy	Median (days) and interquartile range	25	0 (0%)	Median: 40 days IQR: 36 – 46
T-5	Days between last neaodjuvant treatment and surgery (stage III NSCLC)	Patients with NSCLC stage III treated with neoadjuvant systemic treatment who undergone surgery	Median (days) and interquartile range	33	0 (0%)	Median: 46 days IQR: 38 – 61
Stage IVA–IVB NSCLC						
T-6	Days between the first consultation in medical oncology and the start of the first treatment	Patients with NSCLC stage IV who receive systemic treatment	Median (days) and interquartile range	313	0 (0%)	Median: 21 days IQR: 11—33

Outcome quality indicators						
Code	Indicator	Eligibility criteria	Outcome measure	Eligible Patients: n	Missing data: n(%)	Quality indicator result
Stage IA–IIB NSCLC						
O-1	RFS in patients with resected stage I and II NSCLC	Patients with NSCLC stages IA to IIB surgically treated with curative intent	i: RFS rate at 1 year ii: RFS rate at 3 years	77	1 (1.3%)	i: 88% ii: 64.2%
O-2	RFS in resected stage II NSCLC patients receiving adjuvant chemotherapy	Patients with NSCLC stages IIA and IIB who are surgically treated and receive adjuvant chemotherapy	i: RFS rate at 1 year ii: RFS rate at 3 years	30	0 (0%)	i: 93.2% ii: 82.2%
O-3	OS in patients with resected stage I and II NSCLC	Patients with NSCLC stages IA to IIB surgically treated with curative intent	i: OS rate at 1 year ii: OS rate at 3 years	77	0 (0%)	i: 97.4% ii: 86.4%
O-4	OS by stage in patients with stage II NSCLC receiving adjuvant chemotherapy	Patients with NSCLC stages IIA and IIB who are surgically treated and receive adjuvant chemotherapy	i: OS rate at 1 year ii: OS rate at 3 years	30	0 (0%)	i: 100% ii: 96%
Stage IIIA–IIIC NSCLC						
O-5	TTF in patients with stage III NSCLC treated with curative intent	Stage III NSCLC patients who undergo any radical treatment.(surgery or radiotherapy)	IIIA: TTF rate at 1 & 2 years IIIB: TTF rate at 1 & 2 years IIIC: TTF rate at 1 & 2 years	IIIA: 91 IIIB: 41 IIIC: 12	0 (0%)	IIIA: 75.1%; 45.8% IIIB: 68%; 26.9% IIIC: 58.3%; 38.9%
O-6	TTF in patients with stage III NSCLC who receive multimodal treatment including systemic chemotherapy	Stage III NSCLC who receive multimodal treatment including systemic chemotherapy	IIIA: TTF rate at 1 & 2 years IIIB: TTF rate at 1 & 2 years IIIC: TTF rate at 1 & 2 years	IIIA: 80 IIIB: 39 IIIC: 11	0 (0%)	IIIA: 79.3%; 50.4% IIIB: 66.3%; 25.6% IIIC: 63.6%; 42.4%
O-7	OS in patients with stage III NSCLC treated with curative intent	Stage III NSCLC patients who undergo any radical treatment.(surgery or radiotherapy)	IIIA: OS rate at 1 & 3 years IIIB: OS rate at 1 & 3 years IIIC: OS rate at 1 & 3 years	IIIA: 91 IIIB: 41 IIIC: 12	0 (0%)	IIIA: 90.9%; 56.9% IIIB: 85%; 48.7% IIIC: 75%; 64.3%
O-8	OS in patients with stage III NSCLC who receive multimodal treatment including systemic chemotherapy	Stage III NSCLC who receive multimodal treatment including systemic chemotherapy	IIIA: OS rate at 1 & 3 years IIIB: OS rate at 1 & 3 years IIIC: OS rate at 1 & 3 years	IIIA: 80 IIIB: 39 IIIC: 11	0 (0%)	IIIA: 93.5%; 62% IIIB: 84.2%; 48.4% IIIC: 81.2%; 70,1%
Stage IVA–IVB NSCLC						
O-9	OS in patients with stage IV NSCLC with driver mutation treated with target therapy	Stage IV NSCLC patients receiving target therapy	i: OS rate at 1 year ii: OS rate at 2 years iii: OS rate at 3 years	59	0 (0%)	i: 93.2% ii: 70.5% iii: 44.4%
O-10	OS by stage in patients with stage IV NSCLC without driver mutation receiving systemic treatment	Stage IV NSCLC patients receiving systemic therapy	i: OS rate at 1 year ii: OS rate at 2 years iii: OS rate at 3 years	251	0 (0%)	i: 53.3% ii: 36.4% iii: 26.7%
O-11	OS by stage in patients with stage IV NSCLC without driver mutation receiving immunotherapy (any line)	Stage IV NSCLC patients receiving immuntherapy	i: OS rate at 1 year ii: OS rate at 2 years iii: OS rate at 3 years	148	0 (0%)	i: 72% ii: 52.9% iii: 37.2%

*NSCLC* non-small cell lung cancer; *OS* overall survival; *RFS* relapse free survival, *TTF* time to treatment failure

## Discussion

Our study demonstrates that it is possible to extract data from the EPR and calculate QIs automatically using an algorithm built based on the recommendations of clinical practice guidelines in NSCLC. The decision tree programmed for the application of data mining in the EPR allows us to identify the therapeutic journey and perform a quality of care assessment in our NSCLC cohort, resulting in a tool to monitor quality improvement.

The process indicators allow us to evaluate adherence to clinical practice guidelines, observing a higher proportion of patients with stages I–II and stage IIIA treated with surgery than reported in other series such as that of Andreano et al., with 55.4%, 48.3% and 35.8% of patients who undergone surgery on stages I, II and III, respectively [11], or 75.2% in stage I and 56.9% in stage II reported by Guerreiro et al [15]. The utilization of systemic treatment in stage IV patients in our study was 73.4% in non-mutated patients and 96.9% in mutated patients, exceeding the 60% target reported for this indicator [15]. It is also possible to evaluate the implementation over time of new therapies such as checkpoint inhibitors for non-mutated patients in stage IV, confirming a progressive increase from 40.3% in 2016 to 71.2% in 2020.

With the outcome indicators, we try to measure the effectiveness of care, the most relevant being survival [12], however, real-life data on this indicator show great variability for NSCLC [13]. In our study, the 3-year survival rate was 86.4% in stages I and II, 54.9% in stage III, and 44.4% and 26.8% in mutated and non-mutated stage IV patients, respectively, higher proportions than those described in The Surveillance, Epidemiology, and End Results (SEER) program at the same follow-up point and for patients diagnosed in the year 2017: 83.3%, 59.5%, 19.9%, for early stages, locally advanced and advanced disease, respectively among adenocarcinoma patients, and 61%, 35.5%, and 11.1% for the same stage groups among squamous cell carcinoma patients [16]. However, our survival assessment does not seek to compare populations, but rather to contribute to the interpretation of global care for NSCLC, allowing us to explain that the differences observed are due to the selection of the population by the treatment received in each subgroup. Our outcome indicator better reflects the effectiveness of the treatments recommended in the clinical guidelines, having previously identified their correct application in the evaluation of processes.

Regarding the diagnostic process, pathological confirmation was performed in 100% of the patients, the histological subtype was identified in 91.9%, the TNM stage was documented in 100% and the relevant molecular study was available in 94.7% of the patients in stage IV. The completeness of these variables, together with the availability of information on health status such as the ECOG and the Charlson comorbidity index, seeks to resolve the lack of information on patient prognosis, a common limitation of real-world studies to evaluate care and outcomes [6]. We observed a higher proportion of compliance with diagnosis-related QIs compared to other studies, such as 81.8% histological confirmation reported by Beck et al [8], or 83.8% in a large series evaluated in Italy [11]. Also in the documentation of the TNM stage as 88.57% of the work of Mazzone et al. [17], or 89% of patients with molecular study observed in the Dutch Lung Cancer Audit [13].

Among QIs, outcome indicators have always attracted greater interest for both patients and health policy makers [7, 18, 19], However, its interpretation is difficult without understanding the processes that lead to its achievement, the latter being more sensitive to differences in the quality of care as they do not depend on individual factors of the patient or on the unfavorable evolution of advanced cancer even if the correct treatment is applied [19]. Improving the OS of patients with NSCLC requires improving all processes related to their diagnosis and treatment, therefore, we propose a set of quality metrics that encompass all NSCLC care, diagnostic processes, therapeutic processes, and outcomes.

Although having references may be useful to propose quality policies, obtaining QIs for comparison with other studies is not the objective of our study. Comparability of treatments and outcomes between studies or hospitals should take into account differences between populations, potential time bias according to the follow-up period of each cohort, or lack of data that may dictate appropriate treatment. In addition to these limitations, we decided not to use target values for the indicators due to the lack of consensus on them and because the recommendations of the guidelines are only applicable to subgroups of patients, while the QIs have often been calculated in heterogeneous populations. Assessing timely care is even more controversial, with studies associating increased survival with shorter time to treatment [12] and others describing the so-called “wait time paradox”, in which shorter time to care was associated with worse outcomes [15]. This phenomenon has been partly justified by the urgency of treatment in more symptomatic patients, or by the delay in completing molecular studies that could be beneficial for patients. Despite the controversy, timely care is one of the dimensions of quality recognized by the Institute of Medicine and considered valuable to patients [20]. Cross-hospital benchmarking could be one of the potential applications of QIs, but should be used with caution when the reasons for the correct exception to each recommendation cannot be assessed [11].

The quality measures described are considered effective in modifying hospital practices and policies, are useful for tracking improvements, and can increase the value of health care [21], but its implementation has historically been hampered by the administrative workload and time required for data collection [9]. In our study, we overcame these limitations using the CRISP-DM methodology, previously applied to the study of healthcare consumption for lung cancer [14], taking advantage of data mining in the use of available data and saving time in their extraction. CRISP-DM includes six phases: business understanding, data understanding, data preparation, modeling, evaluation, and implementation [22], which are cyclically repeated to improve the process. The use of decision trees in data exploration is especially useful for identifying the care received and its sequence, as it allows logical diagrams to be generated to categorize conditions. A path to be followed is defined depending on the value of each variable considered, until it ends up belonging or not to a group [23, 24], with the added value of offering interpretable visual representation. In our study, we configured an analysis algorithm based on a decision tree that defined the therapeutic routes according to the clinical guidelines, which, by interrogating the set of data generated in routine patient care, proves to be a feasible solution for obtaining indicators.

The methodology used not only solves the question of how to measure quality, but also determines which indicators to measure. A variety of techniques have been used in health care quality assessment studies to develop or select indicators: literature review, expert consensus, or Delphi review among them [4, 18, 25–27]. In our study, the QIs were selected by matching recommendations from clinical practice guidelines with indicators identified in the literature review, a strategy previously used in NSCLC [8, 11–13]. With the use of clinical guidelines, we guarantee the relevance of the selected QIs in the current management of lung cancer. Finally, we assumed as a selection criterion its feasibility of calculation [11, 28], constructing a measurement feedback system using the EPR, previously suggested to solve the slowness and burden of manual data abstraction [8, 9, 29].

The complexity of cancer care requires planning and performance analysis to ensure its alignment with the current state of knowledge and achieve the highest level of quality in the health system [19]. The main implication of our work is to have a tool to respond to this unmet need. Our work proposes a quality assessment procedure, not only for comparative evaluation, but also to monitor continuous quality improvement. As far as we know, this is the first study to evaluate the care provided by a Medical Oncology service to all patients with NSCLC of any stage in Spain. We believe that the use of data automatically collected from the EPR and generated in routine practice allows our methodology to be scalable at the national level.

Our work has several limitations: first, the initial review was carried out by a single oncologist, compared to most studies in which a group of experts participates. We understand that there is enough number of indicators recognized as valid to shorten their discovery and prioritize their applicability. Second, the selection of indicators based on available data may miss important indicators. This should encourage the collection and coding of a minimum dataset needed to monitor care processes, as well as facilitate their automatic acquisition. Finally, real-world data are acquired in an uncontrolled environment, with incorrectly recorded data having been included or resulting in no data at all. Considering this issue, data entry was performed by personnel trained in documentation, consistent with the very low proportion of missing values observed.

## Conclusion

To achieve the highest quality in healthcare, it is necessary to have feasible metrics and measurement tools that monitor actions and continuous changes. Our study demonstrates that it is possible, through the application of an algorithm created based on the recommendations of clinical practice guidelines in NSCLC, to use data generated in routine practice to reconstruct the patient trajectory and evaluate the quality of care provided by a Medical Oncology service through 34 QIs.

The digitalization of healthcare has made it possible to access more data than ever before in the history of medicine. Our work proposes a methodology to take advantage of the data contained in hospital information sources, allowing feedback and repeated measurement over time, thus offering a tool to understand compliance with evidence-based recommendations, monitor interventions and, ultimately, establish a system of continuous improvement of the quality of health care.

## Supplementary Information

Below is the link to the electronic supplementary material.

Supplementary file1 (DOCX 54 KB)

## Data Availability

The availability of data is possible upon request subject to the protocol approved by Institutional Ethics Board.
