# Supplementary material for: Quality of care assessment for non-small cell lung cancer patients: transforming routine care data into a continuous improvement system
Source: Clin Transl Oncol. 2024 Aug 16;27(3):1047–61. doi: 10.1007/s12094-024-03658-3 (PMC11913935; doi:10.1007/s12094-024-03658-3)
Supplement: Supplementary file 1 — Supplementary file1 (DOCX 54 KB) [file 12094_2024_3658_MOESM1_ESM.docx]

**SUPPLEMENTARY APENDIX**

1. **SUPPLEMENTARY METHODS:**
   1. **SELECTION OF QUALITY INDICATORS:**
      1. ***Definition of quality indicator****:*

The definition used by the Spanish Lung Cancer Group (GECP) for quality indicators is assumed: “measurable elements of practice performance for which there is evidence or consensus that they can assess the quality of care provided and the effect of certain changes in it” [Guirado M, 2022].

- - 1. ***Pubmed search strategy:***

("Lung Neoplasms"[MeSH] OR "lung cancer") AND ("Quality Indicators, Health Care"[MeSH] OR "quality metrics" OR "quality of care" OR "quality indicators").

Limited to articles from January 1, 2005, until November 15, 2023, with no language restrictions.

- - 1. ***Grey literature search:***

We identified the most relevant clinical practice guidelines for the management of NSCLC in our center. An evaluation of the recommendations was carried out, selecting the following clinical guidelines for their applicability to the studied population and the feasibility of relating the main recommendations with calculable indicators:

- National Comprehensive Cancer Network (NCCN).
- American Society of Clinical Oncology (ASCO).
- European Society for Medical Oncology (ESMO).
- Sociedad Española de Oncología Médica (SEOM).
- International Association for the Study of Lung Cancer (IASLC).
- British Thoracic Society.
- The National Institute for Health and Care Excellence (NICE).
- European Respiratory Society.
- American Society of Clinical Oncology Quality Oncology Practice Initiative.
- International Consortium for Health Outcomes Measurement (ICHOM).
  - 1. ***Quality indicators selection:***

A total of 128 indicators were selected from an initial literatura review. The indicators obtained were related to clinical practice recommendations, proceeding to their grouping. In cases of minor differences in time windows or in the definition of the denominator, the most suitable one was selected for the database and the care process. We proceeded to evaluate their feasibility of calculation in the available dataset and select their applicability in the context of the previously constructed patient trajectory.

Finally, 34 indicators were selected: 17 process indicators, 6 process indicators that specifically evaluate the timeliness of care, and 11 outcome indicators.

- 1. **DATA SOURCES:**

The following data were obtained from Puerta de Hierro University Hospital cancer registries:

- Data from the HUPHM tumor registry between January 1st 2016 and December 31st 2021: 12,581 records with 40 variables.
- Data from the Medical Oncology Service registry of HUPHM between January 1st 2016 and December 31st 2021: 4,339 records with 73 variables.

The following data were obtained from the data warehouse system of the Electronic Patients Records:

- Data of medical oncology appointments between January 1st 2016 and June 30th 2021: 121,878 records with 20 variables.
- Data of emergency service attendance between January 1st 2016 and June 30th 2021: 881,738 with 33 variables.
- Data of hospital admittances between January 1st 2016 and June 30th 2021 taken from the coded hospital registry through ICD-10: 140,432 records with 138 variables.
- Data of systemic treatments between January 1st 2016 and June 30th 2021 taken from the electronic pharmacy prescription: 62,277 records with 40 variables.
  1. **SURVIVAL OUTCOMES DEFINITIONS:**
- Relapse-free survival (RFS): used in indicator O-1 and O-2, applicable to patients with NSCLC stages I and II. It was defined as the time from the date of surgery with curative intent to the date of the first loco-regional relapse, documented distant relapse, or death from any cause, whichever occurred first. Patients who were event-free at the end of the follow-up were censored on the date of the last follow-up.
- Time to treatment failure (TTF): used in indicator O-5 and O-6, applicable to stage III patients treated with some form of radical treatment, surgery, or radiotherapy. It was defined as the time from diagnosis to the date of the first loco-regional relapse, locoregional progression in case of incomplete response to radiotherapy, documented distant relapse, or death from any cause, whichever occurred first. Patients who were event-free at the end of the follow-up were censored on the date of the last follow-up.
- Overall survival (OS) by stage: used in indicator O-3, O-4, O-7, O-8, O-9, O-10, and O-11, applicable to all patients with NSCLC but studied according to the stage. It was defined as the time from the date of diagnosis to the date of death from any cause. Patients who were event-free at the end of the follow-up were censored on the date of the last follow-up.

1. **SUPPLEMENTARY RESULTS:**
   1. **SURVIVAL ANALYSIS:**

We report the results of the survival analysis for outcomes indicators, adding supplementary information on medians and 95% confidence interval, survival rate at 6 months, 1, 2, 3 and 4 years.

| **O1: RELAPSE FREE SURVIVAL IN PATIENTS WITH RESECTED STAGE I AND II NSCLC.** | | | | | | |
| --- | --- | --- | --- | --- | --- | --- |
| **Stage (n)** | **IA** (18) | **IB** (22) | **IIA** (15) | **IIB** (29) | **Total NSCLC I-II** | ***p*** |
| **Resected NSCLC: n** | 17 | 18 | 15 | 27 | 77 | 0.220 |
| **Relapse or death: n (%)** | 4 (23.4%) | 7 (38.9%) | 3 (20%) | 7 (25.9%) | 21 (27.9%) | 0.466 |
| **Median RFS: months (95%CI)** | NR (27.9 - NR) | 53,1 (13.8 - NR) | NR (18.8 - NR) | NR (25.4 - NR) | NR (53.1 - NR) | Log-rank: 0.515 |
| **RFS rate:** |  | | | | | |
| **6 months** | 100% | 88.9%  (62.4% - 97.1%) | 93.3%  (61.3% - 99%) | 88.5%  (68.4% - 96.1%) | 92.1%  (83.3% - 96.4%) |  |
| **12 months** | 100% | 83.3%  (56.8% - 94.3%) | 86.7%  (56.4% - 96.5%) | 84.3%  (63.2% - 93.8%) | 88%  (78.3% - 93.6%) |  |
| **24 months** | 85.2%  (51.9% - 96.2%) | 70.5%  (42.8% - 86.6%) | 78.8%  (47.3% - 92.7%) | 75.6%  (53.4% - 88.3%) | 77.3%  (65.5% - 85.5%) |  |
| **36 months** | 64.9%  (30.3% - 85.6%) | 54.4%  (26.2% - 75.8%) | 78.8%  (47.3% - 92.7%) | 62%  (36.3% - 79. 8%) | 64.2%  (50.1% - 75.3%) |  |
| **48 months** | 64.9%  (30.3% - 85.6%) | 54.4%  (26.2% - 75.8%) | 78.8%  (47.3% - 92.7%) | 62%  (36.3% - 79.8%) | 64.2%  (50.1% - 75.3%) |  |
|  | | | | | | |

| **O2: RELAPSE FREE SURVIVAL IN RESECTED STAGE II NSCLC PATIENTS RECEIVING ADJUVANT CHEMOTHERAPY.** | | | | |
| --- | --- | --- | --- | --- |
| **Resected stage II NSCLC (n)** | **IIA** (15) | **IIB** (27) | **Total NSCLC II** (42) | ***p*** |
| **Adjuvant treatment: n** | 10 | 20 | 30 | 0.611 |
| **Relapse or death: n (%)** | 2 (20%) | 5 (25%) | 7 (23.3%) | 0.760 |
| **Median RFS: months (95%CI)** | NR (8.7 - NR) | NR (25.4 - NR) | NR (NR - NR) | Log-rank: 0.618 |
| **RFS rate:** |  | | | |
| **6 months** | 100% | 95%  (69.5% - 99.3%) | 96.7%  (78.6% - 99.5%) |  |
| **12 months** | 90%  (47.3% - 98.5%) | 95%  (69.5% - 99.3%) | 93.2%  (75.5% - 98.3%) |  |
| **24 months** | 80%  (40.9% - 94.6%) | 83.5%  (56.8% - 94.4%) | 85.9%  (66.6% - 94.5%) |  |
| **36 months** | 80%  (40.9% - 94.6%) | 66.9%  (36.1% - 85.4%) | 82.2%  (62.2% - 92.2%) |  |
| **48 months** | 80%  (40.9% - 94.6%) | 66.9%  (36.1% - 85.4%) | 72.7%  (50.4% - 86.2%) |  |
|  | | | | |

| **O3: OVERALL SURVIVAL IN PATIENTS WITH RESECTED STAGE I AND II NSCLC.** | | | | | | |
| --- | --- | --- | --- | --- | --- | --- |
| **Stage (n)** | **IA** (18) | **IB** (22) | **IIA** (15) | **IIB** (29) | **Total NSCLC I-II** | ***p*** |
| **Resected NSCLC: n** | 17 | 18 | 15 | 27 | 77 | 0.220 |
| **Death: n (%)** | 2 (11.8%) | 3 (16.7%) | 1 (6.7%) | 3 (11.1%) | 9 (11.7%) | 0.848 |
| **Median RFS: months (95%CI)** | NR (28.2 - NR) | NR (36.8 - NR) | NR (24.2 - NR) | NR (NR - NR) | NR (NR - NR) | Log-rank: 0.016 |
| **RFS rate:** |  | | | | | |
| **6 months** | 100% | 100% | 100% | 96.3%  (76.5% - 99.5%) | 98.7%  (91.1% - 99.8%) |  |
| **12 months** | 100% | 100% | 100% | 92.6%  (73.5% - 98.1%) | 97.4%  (90% - 99.3%) |  |
| **24 months** | 100% | 93.8%  (63.2% - 99.1%) | 100% | 88%  (66.9% - 96%) | 94.3%  (85.4% - 97.8%) |  |
| **36 months** | 83.3%  (48.2% - 95.6%) | 85.2%  (51.9% - 96.2%) | 90%  (47.3% - 98.5%) | 88%  (66.9% - 96%) | 86.4%  (74.2% - 93.1%) |  |
| **48 months** | 83.3%  (48.2% - 95.6%) | 76.7%  (43.1% - 92%) | 90%  (47.3% - 98.5%) | 88%  (66.9% - 96%) | 83.9%  (70.8% - 91.5%) |  |
|  | | | | | | |

| **O4: OVERALL SURVIVAL IN RESECTED STAGE II NSCLC PATIENTS RECEIVING ADJUVANT CHEMOTHERAPY.** | | | | |
| --- | --- | --- | --- | --- |
| **Resected stage II NSCLC (n)** | **IIA** (15) | **IIB** (27) | **Total NSCLC II** (42) | ***p*** |
| **Adjuvant treatment: n** | 10 | 20 | 30 | 0.611 |
| **Death: n (%)** | 1 (10%) | 0 (0%) | 1 (3.3%) | 0.150 |
| **Median OS: months (95%CI)** | NR (24.2 - NR) | NR (NR - NR) | NR (NR - NR) | Log-rank: 0.182 |
| **OS rate:** |  | | | |
| **6 months** | 100% | 100% | 100% |  |
| **12 months** | 100% | 100% | 100% |  |
| **24 months** | 100% | 100% | 100% |  |
| **36 months** | 88.9%  (43.3% - 98.4%) | 100% | 96%  (74.8% - 99.4%) |  |
| **48 months** | 88.9%  (43.3% - 98.4%) | 100% | 96%  (74.8% - 99.4%) |  |
|  | | | | |

| **O5: TIME TO TREATMENT FAILURE IN PATIENTS WITH STAGE III NSCLC TREATED WITH CURATIVE INTENT.** | | | | | |
| --- | --- | --- | --- | --- | --- |
| **Stage (n)** | **IIIA** (94) | **IIIB** (52) | **IIIC** (14) | **Total NSCLC III** (160) | ***p*** |
| **Surgery or RT: n** | 91 | 41 | 12 | 144 | ***0.002*** |
| **Relapse or death: n (%)** | 51 (56%) | 33 (80.5%) | 8 (66.7%) | 92 (63.9%) | ***0.025*** |
| **Median TTF: months (95%CI)** | 21.7 (16.2 – 38.2) | 13.6 (12.3 – 17.8) | 21.4 (7.3 – NR) | 17.6 (15.1 – 22.5) | Log-rank: ***0.042*** |
| **TTF rate:** |  | | | | |
| **6 months** | 95.6%  (88.7% - 98.3%) | 95.1%  (81.9% - 98.8%) | 100% | 95.8%  (91% - 98.1%) |  |
| **12 months** | 75.1%  (64.7% - 82.9%) | 68%  (51.3% - 80%) | 58.3%  (27% - 80.1%) | 71.6%  (63.3% - 78.3%) |  |
| **24 months** | 45.8%  (34.6% - 56.3%) | 26.9%  (14.2% - 41.3%) | 38.9%  (12.6% - 65%) | 39.5%  (31.1% - 47.9%) |  |
| **36 months** | 39.3%  (28.3% - 50.2%) | 21.2%  (9.9% - 35.2%) | - | 32.7%  (24.5% - 41.1%) |  |
| **48 months** | 37.4%  (26.3% - 48.4%) | 15.9%  (5.5% - 31.1%) | - | 29.6%  (21.3% - 38.4%) |  |
|  | | | | | |

| **O6: TIME TO TREATMENT FAILURE IN PATIENTS WITH STAGE III NSCLC TREATED WITH A MULTIMODAL TREATMENT STRATEGY INCLUDING SYSTEMIC CHEMOTHERAPY.** | | | | | |
| --- | --- | --- | --- | --- | --- |
| **Stage (n)** | **IIIA** (94) | **IIIB** (52) | **IIIC** (14) | **Total NSCLC III** (160) | ***p*** |
| **Multimodal therapy: n** | 80 | 39 | 11 | 130 | 0.314 |
| **Relapse or death: n (%)** | 42 (52.5%) | 31 (79.5%) | 7 (63.6%) | 80 (61.5%) | ***0.018*** |
| **Median TTF: months (95%CI)** | 24.5 (17.2 – NR) | 13.6 (11.4 – 17.8) | 22.5 (9.2 – NR) | 20.1 (15.8 – 24.5) | Log-rank: ***0.011*** |
| **TTF rate:** |  | | | | |
| **6 months** | 96.3%  (88.8% - 98.8%) | 94.9%  (81% - 98.7%) | 100% | 96.2%  (91% - 98.4%) |  |
| **12 months** | 79.3%  (68.5% - 86.8%) | 66.3%  (49.1% - 78.9%) | 63.6%  (29.7% - 84.5%) | 73.9%  (65.4% - 80.7%) |  |
| **24 months** | 50.4%  (38.3% - 61.4%) | 25.6%  (13% - 40.3%) | 42.4%  (13.7% - 69.1%) | 41.9%  (32.9% - 50.7%) |  |
| **36 months** | 43.3%  (31.2% - 54.8%) | 19.5%  (8.5% - 33.8%) | - | 34.5%  (25.7% - 43.4%) |  |
| **48 months** | 41.1%  (29% - 52.8%) | 13%  (3.3% - 29.4%) | - | 31%  (22.1% - 40.4%) |  |
|  | | | | | |

| **O7: OVERALL SURVIVAL IN PATIENTS WITH STAGE III NSCLC TREATED WITH CURATIVE INTENT.** | | | | | |
| --- | --- | --- | --- | --- | --- |
| **Stage (n)** | **IIIA** (94) | **IIIB** (52) | **IIIC** (14) | **Total NSCLC III** (160) | ***p*** |
| **Surgery or RT: n** | 91 | 41 | 12 | 144 | ***0.002*** |
| **Death: n (%)** | 34 (37.4%) | 20 (48.8%) | 5 (41.8%) | 59 (41%) | 0.466 |
| **Median OS: months (95%CI)** | NR (31.6 – NR) | 30.1 (21.8 – NR) | 36.5 (11.2 – NR) | 52.8 (30.1 – NR) | Log-rank: 0.565 |
| **OS rate:** |  | | | | |
| **6 months** | 97.8%  (91.5% - 99.5%) | 97.6%  (83.9% - 99.7%) | 100% | 97.9%  (93.7% - 99.3%) |  |
| **12 months** | 90.9%  (82.6% - 95.3%) | 85%  (69.5% - 93%) | 75%  (40.8% - 91.2%) | 87.8%  (81.1% - 92.2%) |  |
| **24 months** | 71.9%  (60.7% - 80.4%) | 61.8%  (43.8% - 75.5%) | 75%  (40.8% - 91.2%) | 69.2%  (60.4% - 76.5%) |  |
| **36 months** | 56.9%  (44.3% - 67.6%) | 48.7%  (31% - 64.2%) | 64.3%  (29.8% - 85.1%) | 54.9%  (45.1% - 63.6%) |  |
| **48 months** | 52.6%  (39.7% - 64%) | 48.7%  (31% - 64.2%) | - | 50.7%  (29.3% - 55.2%) |  |
|  | | | | | |

| **O8: OVERALL SURVIVAL IN PATIENTS WITH STAGE III NSCLC WHO RECEIVE MULTIMODAL TREATMENT INCLUDING SYSTEMIC CHEMOTHERAPY.** | | | | | |
| --- | --- | --- | --- | --- | --- |
| **Stage (n)** | **IIIA** (94) | **IIIB** (52) | **IIIC** (14) | **Total NSCLC III** (160) | ***p*** |
| **Multimodal strategy: n** | 80 | 39 | 11 | 130 | 0.314 |
| **Death: n (%)** | 26 (32.5%) | 18 (46.2%) | 4 (36.4%) | 48 (36.9%) | 0.350 |
| **Median OS: months (95%CI)** | NR (34 – NR) | 30.1 (21.8 – NR) | 36.5 (11.9 – NR) | 55.5 (11.9 – NR) | Log-rank: 0.302 |
| **OS rate:** |  | | | | |
| **6 months** | 98.8%  (91.5% - 99.8%) | 97.4%  (83.2% - 99.6%) | 100% | 98.5%  (94% - 99.6%) |  |
| **12 months** | 93.5%  (84.9% - 97.2%) | 84.2%  (68.1% - 92.6%) | 81.2%  (44.7% - 95.1%) | 89.6%  (82.7% - 93.8%) |  |
| **24 months** | 78.9%  (67.3% - 86.7%) | 62.3%  (43.7% - 76.3%) | 81.2%  (44.7% - 95.1%) | 74.1%  (65% - 81.2%) |  |
| **36 months** | 62%  (48.3% - 73%) | 48.4%  (30.2% - 64.4%) | 70.1%  (32.3% - 89.5%) | 58.3%  (47.9% - 67.3%) |  |
| **48 months** | 57.2%  (43.1% - 69%) | 48.4%  (30.2% - 64.4%) | - | 53.6%  (42.8% - 63.3%) |  |
|  | | | | | |

| **O9: OVERALL SURVIVAL IN PATIENTS WITH STAGE IV NSCLC WITH DRIVER MUTATION TREATED WITH TARGET THERAPY.** | | | | |
| --- | --- | --- | --- | --- |
| **Stage: n** | **IVA with driver mutation** (20) | **IVB with driver mutation** (44) | **NSCLC IV with driver mut.** (64) | ***p*** |
| **Target therapy: n** | 17 | 42 | 59 |  |
| **Death: n (%)** | 9 (52.9%) | 19 (45.2%) | 28 (47.5%) |  |
| **Median OS: months (95%CI)** | 48.2 (21.5 – NR) | 34.9 (23.5 – NR) | 34.9 (26 – 50.1) | Log-rank: 0.707 |
| **OS rate:** |  | | | |
| **6 months** | 88.2%  (60.6% - 96.9%) | 95.2%  (82.3% - 98.8%) | 93.2%  (82.9% - 97.4%) |  |
| **12 months** | 88.2%  (60.6% - 96.9%) | 95.2%  (82.3% - 98.8%) | 93.2%  (82.9% - 97.4%) |  |
| **24 months** | 80.2%  (49.6% - 93.3%) | 67%  (48.9% - 80%) | 70.5%  (55.6% - 81.3%) |  |
| **36 months** | 53.5%  (23.4% - 76.4%) | 39.6%  (20.3% - 58.4%) | 44.4%  (28.2% - 59.4%) |  |
| **48 months** | 53.5%  (23.4% - 76.4%) | 39.6%  (20.3% - 58.4%) | 44.4%  (28.2% - 59.4%) |  |
|  | | | | |

| **O10: OVERALL SURVIVAL IN PATIENTS WITH STAGE IV NSCLC WITHOUT DRIVER MUTATION RECEIVING SYSTEMIC TREATMENT.** | | | | |
| --- | --- | --- | --- | --- |
| **Stage: n** | **IVA without driver mutation** (128) | **IVB without driver mutation** (214) | **NSCLC IV without mutation** (342) | ***p*** |
| **Systemic therapy: n** | 91 | 160 | 251 | 0.457 |
| **Death: n (%)** | 59 (64.8%) | 122 (76.3%) | 181 (72.1%) | 0.053 |
| **Median OS: months (95%CI)** | 22.5 (15 – 35.3) | 10.3 (7.8 – 13.2) | 13.6 (10.5 – 17.9) | Log-rank: ***0.002*** |
| **OS rate:** |  | | | |
| **6 months** | 86.8%  (77.9% - 92.3%) | 66.6%  (58.7% - 73.4%) | 74%  (68% - 79%) |  |
| **12 months** | 66.6%  (55.8% - 75.3%) | 45.7%  (37.7% - 53.3%) | 53.3%  (46.9% - 59.3%) |  |
| **24 months** | 48.5%  (37.4% - 58.8%) | 29.5%  (22.2% - 37.1%) | 36.4%  (30.2% - 42.7%) |  |
| **36 months** | 35.7%  (24.9% - 46.7%) | 21.6%  (14.8% - 29.2%) | 26.7%  (20.8% - 33.1%) |  |
| **48 months** | 23.5%  (13.2% - 35.6%) | 16.2%  (9.9% - 24%) | 18.9%  (13.2% - 25.4%) |  |
|  | | | | |

| **O11: OVERALL SURVIVAL IN PATIENTS WITH STAGE IV NSCLC WITHOUT DRIVER MUTATION RECEIVING IMMUNOTHERAPY.** | | | | |
| --- | --- | --- | --- | --- |
| **Stage: n** | **IVA without driver mutation** (128) | **IVB without driver mutation** (214) | **NSCLC IV without mutation** (342) | ***p*** |
| **Immunotherapy: n** | 59 | 89 | 148 | 0.416 |
| **Death: n (%)** | 32 (54.2%) | 57 (64%) | 89 (60.1%) | 0.233 |
| **Median OS: months (95%CI)** | 32.7 (23.7 – 47.1) | 19.4 (15.2 – 27.2) | 25.7(18.9 – 32.7) | Log-rank: 0.142 |
| **OS rate:** |  | | | |
| **6 months** | 91.5%  (80.8% - 96.4%) | 88.7%  (80.1% - 93.8%) | 89.9%  (83.7% - 93.8%) |  |
| **12 months** | 76.1%  (63% - 85.1%) | 69.3%  (58.6% - 77.8%) | 72%  (64% - 78.6%) |  |
| **24 months** | 63.6%  (49.2% - 74.9%) | 46%  (34.8% - 56.4%) | 52.9%  (44% - 60.9%) |  |
| **36 months** | 42.4%  (27.4% - 56.8%) | 33.9%  (23% - 45.1%) | 37.2%  (28.2% - 46.2%) |  |
| **48 months** | 32.8%  (17.2% - 49.3%) | 27%  (16.4% - 38.7%) | 29.4%  (20.3% - 39%) |  |
|  | | | | |
